# Neurotoxicity of Perfluorooctane Sulfonate to Hippocampal Cells in Adult Mice

**DOI:** 10.1371/journal.pone.0054176

**Published:** 2013-01-30

**Authors:** Yan Long, Yubang Wang, Guixiang Ji, Lifeng Yan, Fan Hu, Aihua Gu

**Affiliations:** 1 Department of Pharmacology, China Pharmaceutical University, Nanjing, China; 2 The Safety Assessment and Research Center for Drugs of Jiangsu Province, School of Public Health, Nanjing Medical University, Nanjing, China; 3 Key Laboratory of Modern Toxicology of Ministry of Education, School of Public Health, Nanjing Medical University, Nanjing, China; 4 Nanjing Institute of Environmental Sciences/Key Laboratory of Pesticide Environmental Assessment and Pollution Control, Ministry of Environmental Protection, Nanjing, China; 5 State Key Laboratory of Reproductive Medicine, Institute of Toxicology, Nanjing Medical University, Nanjing, China; Institute of Zoology, Chinese Academy of Sciences, China

## Abstract

Perfluorooctane sulfonate (PFOS) is a ubiquitous pollutant and found in the environment and in biota. The neurotoxicity of PFOS has received much concern among its various toxic effects when given during developing period of brain. However, little is known about the neurotoxic effects and potential mechanisms of PFOS in the mature brain. Our study demonstrated the neurotoxicity and the potential mechanisms of PFOS in the hippocampus of adult mice for the first time. The impairments of spatial learning and memory were observed by water maze studies after exposure to PFOS for three months. Significant apoptosis was found in hippocampal cells after PFOS exposure, accompanied with a increase of glutamate in the hippocampus and decreases of dopamine (DA) and 3,4-dihydrophenylacetic acid (DOPAC) in Caudate Putamen in the 10.75 mg/kg PFOS group. By two-dimensional fluorescence difference in gel electrophoresis (2D-DIGE) analysis, seven related proteins in the hippocampus that responded to PFOS exposure were identified, among which, Mib1 protein (an E3 ubiquitin-protein ligase), Herc5 (hect domain and RLD 5 isoform 2) and Tyro3 (TYRO3 protein tyrosine kinase 3) were found down-regulated, while Sdha (Succinate dehydrogenase flavoprotein subunit), Gzma (Isoform HF1 of Granzyme A precursor), Plau (Urokinase-type plasminogen activator precursor) and Lig4 (DNA ligase 4) were found up-regulated in the 10.75 mg/kg PFOS-treated group compare with control group. Furthermore, we also found that (i) increased expression of caspase-3 protein and decreased expression of Bcl-2, Bcl-XL and survivin proteins, (ii) the increased glutamate release in the hippocampus. All these might contribute to the dysfunction of hippocampus which finally account for the impairments of spatial learning and memory in adult mice.

## Introduction

Perfluorinated compounds (PFCs) are persistent, bioaccumulative toxicants. Widespread human exposures to PFCs, including in fetuses, is well documented [1], [2]. Among these compounds, Perfluorooctane sulfonate (PFOS) is the most intensively studied member of PFC family, and is daily used in clothing, carpets, textiles, upholstery, paper, packaging and cleaning products [3]. In recent years, researchers have reported PFCs contamination in river, tap and bottled water in Japan, the US, Europe and in developing countries such as Thailand, Malaysia and Vietnam.

The accumulation of PFOS in mammals is mainly in the liver and serum, as well as in the brain up to as high as 32% of the serum concentration [4]. For PFOS might cross the placenta barrier [5], [6] and blood-brain barrier [7], some previous studies have focused on the developmental neurotoxicity induced by PFOS [8], [9], [10]. Additionally, Johansson et al. found that neonatal exposure of mice to PFOS altered the expression of proteins critical for normal brain development and caused neurobehavioral defects, which persists into adulthood life [11], [12].

A study based on 4,943 mother-child pairs has looked into the relationship between PFOS serum concentration of both child and mother in paired samples over a wide range of the child’s age (1–19 years) and found PFOS concentration tended to be higher in children than in their mothers. Since this difference persisted until they were at least 19 years of age for PFOS [13], it is also important to explore the neurotoxicity of PFOS in adults. The effects of PFOS on adult brain and its potential mechanism remains unclear. It is reported that PFOS exposure cause a deficit in spatial memory in adult male mice [14] without disturbing motor and sensory function, general activity and exploratory behavior. All these indicate that PFOS probably causes deficits in some brain areas such as hippocampus, which is structure directly responsible for the acquisition and the retention of spatial memory and especially vulnerable to damage [15].

The purpose of this study is to determine the neurotoxicity of PFOS treatment and the potential mechanism in adult mice. Herein, the water maze study is utilized to assess impairments in spatial learning and memory after exposure to PFOS for three months. The apoptosis profile of hippocampal cells as well as the levels of glutamate, gamma-aminobutyric acid (GABA), dopamine (DA), 3,4-dihydrophenylacetic acid (DOPAC), and homovanillic acid (HVA) are evaluated (Figure S1. and Figure S2). By two-dimensional fluorescence difference in gel electrophoresis (2D-DIGE) and western blotting analysis, the target proteins in the hippocampus that responded to PFOS exposure are identified to determine potential neurotoxicity of PFOS and its underlying mechanism.

## Results

### Impairment of Spatial Learning and Memory

Hippocampus-dependent spatial learning was tested using the hidden-platform version of the Morris water maze. During the spatial memory task in the water maze, the mice were subjected to 1 daily session for 3 days. On each day, the mice were subjected to 4 acquisition trials during which the hidden platform was located in a fixed position. The escape latency of the control group exhibited decline, while the latency did not significantly change in the groups exposed to 2.15 and 10.75 mg/kg PFOS on the second day.

**Figure 1:**
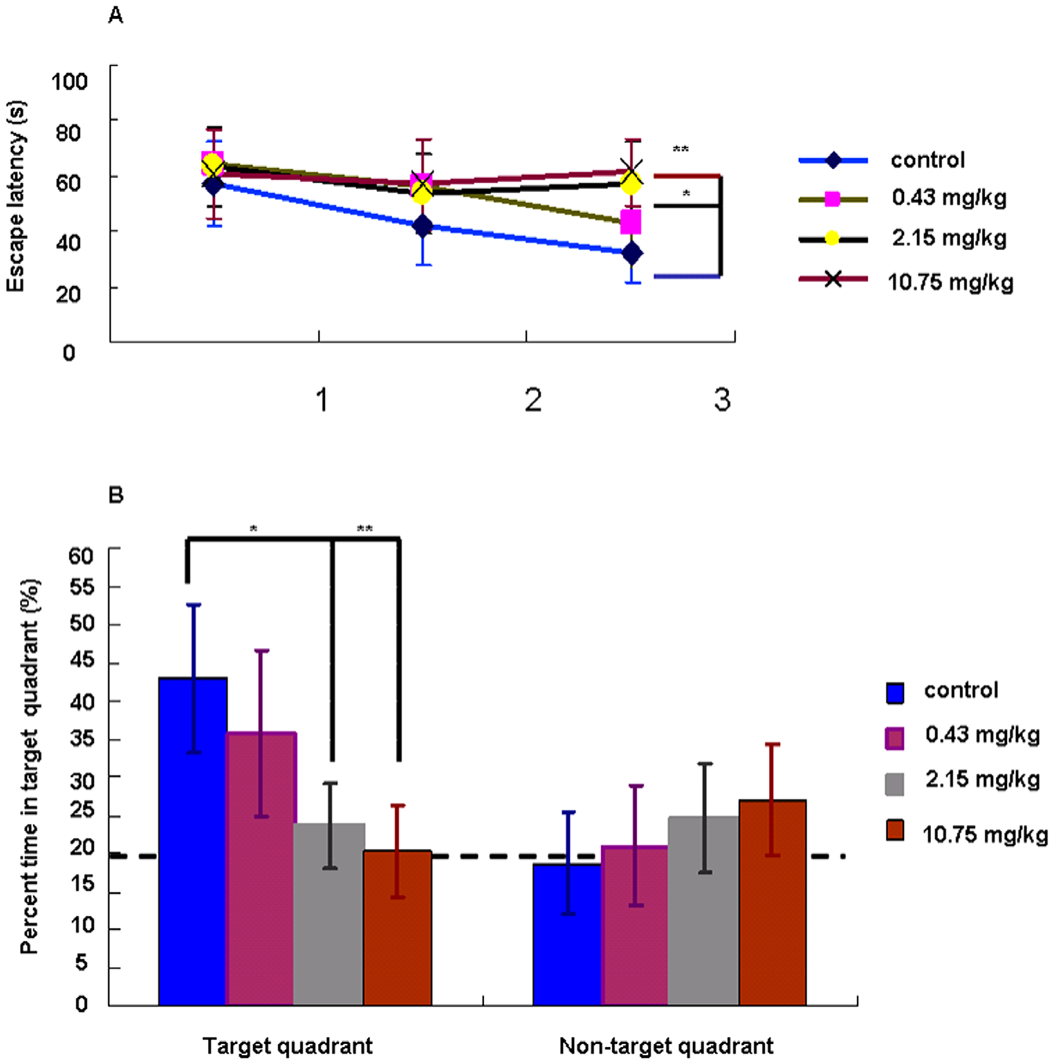
Acquisition and retention of the spatial reference memory task. *(A)* Acquisition of the reference memory task as expressed by the escape latency (mean ± SD, recorded in seconds) over 3 training sessions (four trials/day). Animals treated with 2.15 and 10.75 mg/kg of PFOS exhibited a delayed acquisition compared to animals in the control group. *(B)* The percentage of time spent in the target quadrant relative to the other three quadrants was recorded on the last day. A great retention of the platform location in the 2.15 and 10.75 mg/kg PFOS groups. Mice: n = 15/group. ^*^P<0.05, ^**^P<0.01.

On the third day, the escape latency in the 2.15 mg/kg (56.75±15.57, p<0.05) and 10.75 mg/kg (61.5±12.11, p<0.001) of PFOS-treated groups was significantly decreasedcompared with the control group (32.5±10.69) (Fig. 1A).

Probe trials were performed with the platform removed, which showed the significantly decreased time course percentage spending in the target quadrant in both 2.15 and 10.75 mg/kg groups compared with the control group (for 2.15 mg/kg group, p<0.05; for 10.75 mg/kg group, p<0.01) (Fig. 1B).

In both experiments, mice exhibiting poor swimming velocity, defined as less than 5 cm/s during more than half of the total swim time were excluded from the analysis. Furthermore, no significant difference was found between male and female mice.

### Exposure to PFOS Caused Apoptosis in Hippocampal Cells

**Figure 2:**
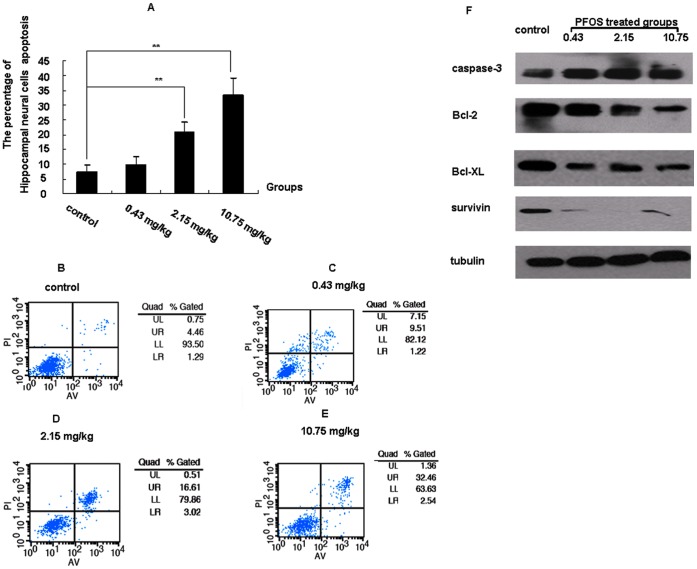
Hippocampal apoptosis neural cells detected by flow cytometry analysis and the related apoptosis proteins expression detected by WB. *(A)* Isolated hippocampal cells were from four groups and stained with annexin-V and PI. Each group of treated cells was analyzed by flow cytometry. The percentage of apoptotic cells was counted as Lower right (LR) plus Upper right quadrant (UR) and expressed as the means ± SD from 10 mice in each group. (B–E) Schematic diagrams of apoptotic hippocampal cells in the control groups and the three PFOS-exposed groups detected by flow cytometry. Representative profiles of hippocampal cells apoptosis in the control group (B), the 0.43 mg/kg group (C), 2.15 mg/kg group (D) and 10.75 mg/kg PFOS-treated groups (E). (F) The expression of caspase-3 was increased while the levels of Bcl-2, Bcl-XL and survivin were decreased in the hippocampus due to PFOS exposure. Mice: n = 10/group. ^**^P<0.01.

By flow cytometry analysis, the apoptosis of hippocampal neural cells was observed in both 2.15 and 10.75 mg/kg PFOS groups. As shown in Fig. 2, the percentage of apoptotic cells in hippocampus was 7.45±2.0 in control group, while were 9.92±2.51, 20.70±3.56 and 33.49±5.77 respectively in the 0.43, 2.15 and 10.75 mg/kg PFOS groups (Fig. 2A–E).

Further studies investigated the apoptosis related proteins in hippocampus, which showed that, in the 2.15 and 10.75 mg/kg PFOS groups, the expression of caspase-3 protein was increased significantly but the expressions of Bcl-2 and survivin proteins were significantly decreased compared with the control group. The decreased expression of Bcl-XL was found only in 10.75 mg/kg PFOS group (Fig. 2F).

### Levels of Neurotrasmitters in Caudate Putamen and Hippocampus

**Figure 3:**
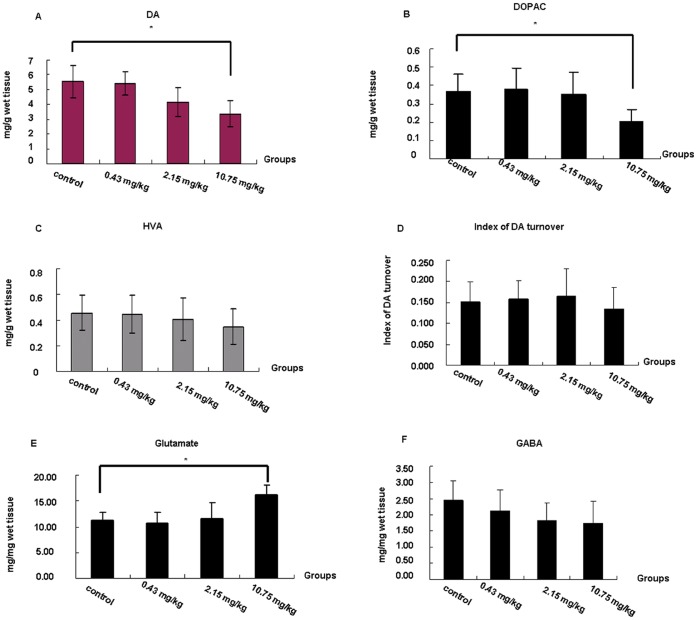
Detection of neurotransmitters release in hippocampus after PFOS exposure. (A–D) DA, DOPAC and HVA release alterations and turnover index for DA in the Caudate Putamen of adult mice exposed to PFOS. Levels of DA *(A)*, DOPAC *(B)*, and HVA *(C)* in the Caudate Putamen of adult mice exposed to PFOS for 3 months. *(D)* Turnover index defined as the (DOPAC+HVA)/DA levels. (E–F) Glutamate and GABA released in hippocampus. Data are expressed as means ± SD (pg/g wet weight of tissue) for 10 animals in each group. ^*^P<0.05 (significant difference from control).

As shown in Figure 3A, although the DA levels was not change obviously in the caudate putamen in the 0.43 and 2.15 mg/kg groups, it decreased significantly in the 10.75 mg/kg PFOS-exposed group compared with the control (p<0.05). Similar results were observed for evaluating the DOPAC level in the caudate putamen which showed a significant decrease in the 10.75 mg/kg of PFOS-treated group (Fig. 3B). While no significant differences were found about the HVA levels (Fig. 3C ) between the control and three PFOS doses. The DA index of turnover, calculated as the ratio of metabolites DOPAC and HVA to DA, was analysed to determine the function in the striatum. The results showed no difference between the PFOS-exposed groups and the control group (Fig. 3D).

Based on the analysis of glutamate level in the hippocampus, a significant increase was found in mice of 10.75 mg/kg PFOS-exposed group compared with those of the control group (Fig. 3E, p<0.05). Although without significance, we also observed that GABA level of PFOS-exposed groups increased slightly compared with that of control group (Fig. 3F).

### Identification of Proteins Differentially Expressed in the PFOS-exposed Mouse Hippocampus

**Figure 4:**
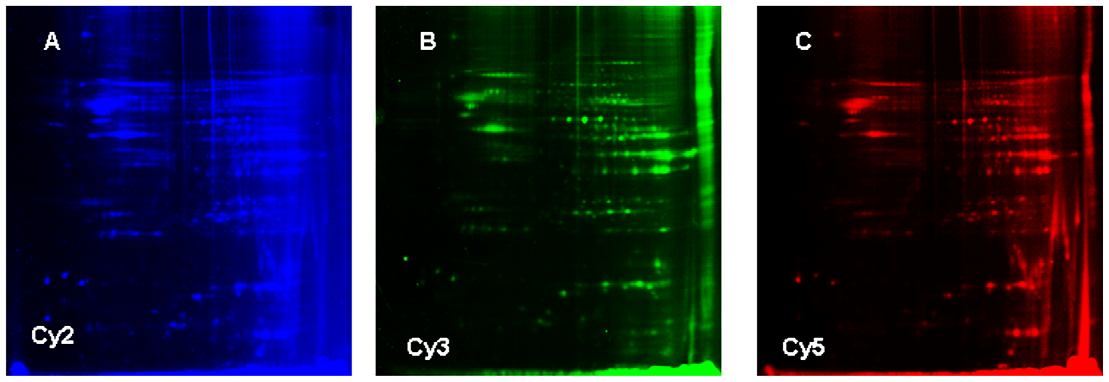
Protein separation, staining and comparison determined by 2D-DEGE. The protein extracts from hippocampus of the control group and 10.75 mg/kg groups were profiled using 2D-DIGE analysis as described in the Materials and methods. (A) Cy2 labeled (internal standard) proteins separated on a 2D-IEF-SDS-PAGE gel, (B) Cy3 labeled proteins from hippocampus of control group, separated on a 2D-IEF-SDS-PAGE gel, (C) Cy5 labedled proteins from hippocampus of 10.75 mg/kg group, separated on a 2D-IEF-SDS-PAGE gel.

**Figure 5:**
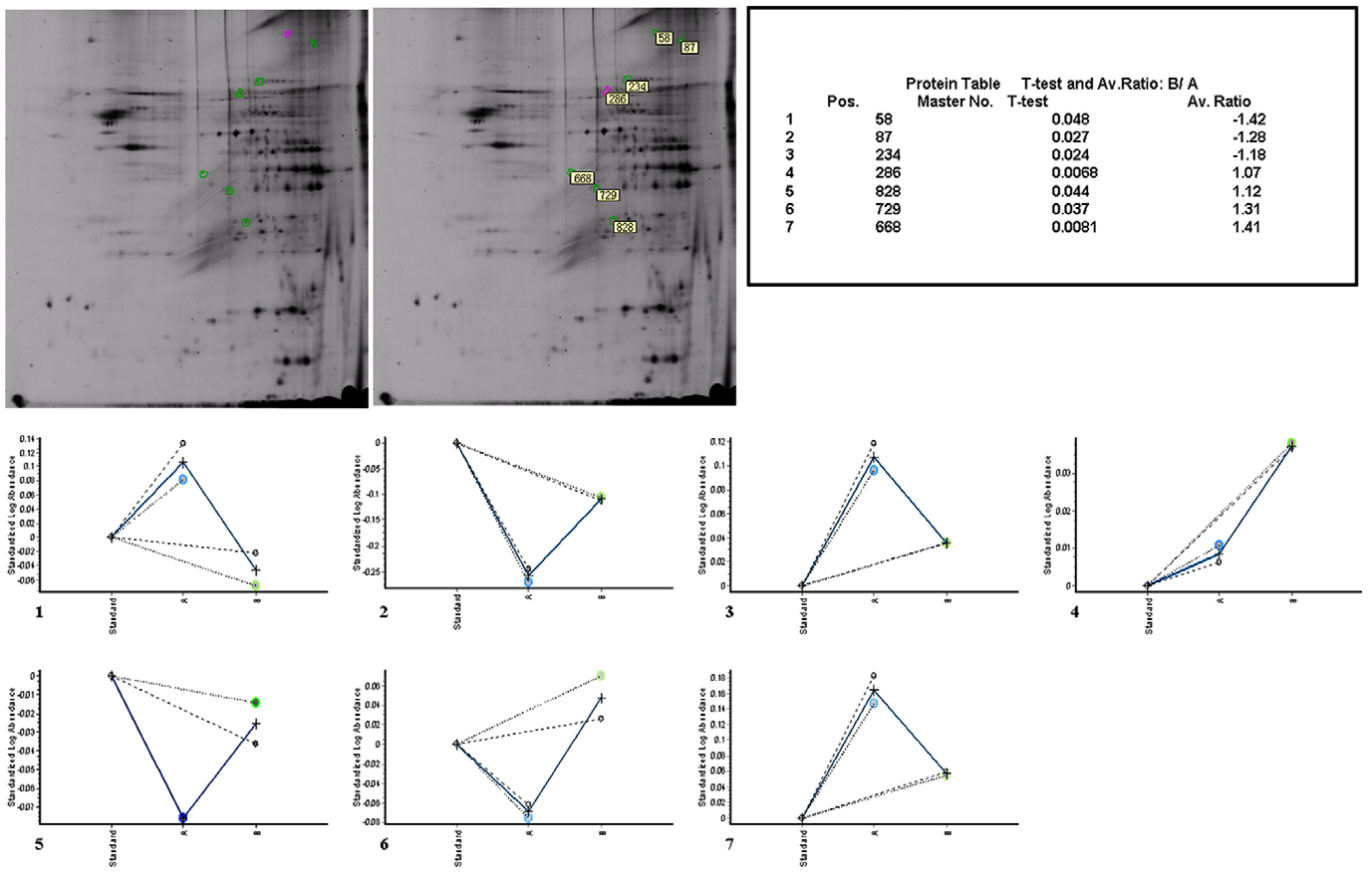
Protein spots identified by LC/MS/MS spectrometer. Target proteins that differentially expressed in adult mice hippocampus after PFOS exposure separated on a 2D-IEF-SDS-PAGE gel and identified by MALDI-TOF MS analysis. The changes were statistically significant at P<0.05. IEF, isoelectric focusing; SDS-PAGE, sodium dodecyl sulfate polyacrylamide gel electrophoresis; 2D, two-dimensional.

Seven differentially expressed proteins were identified by MALDI-TOF MS analysis (Fig. 4, Fig. 5, and Table 1). Among which, Mib1 protein (an E3 ubiquitin-protein ligase), Herc5 (hect domain and RLD 5 isoform 2) and Tyro3 (TYRO3 protein tyrosine kinase 3) were found down-regulated and Sdha (Succinate dehydrogenase flavoprotein subunit), Gzma (Isoform HF1 of Granzyme A precursor), Plau (Urokinase-type plasminogen activator precursor) and Lig4 (DNA ligase 4) were up-regulated after PFOS exposure (10.75 mg/kg group).

**Table 1:** Proteins identified by MALDI-TOF MS analysis.

Master No.	Gene Symbol	Species	Regulated	SWISS-PROT
828	Plau (Urokinase-type plasminogen activator precursor)	Mus Musculus	up-regulated	P06869
286	Lig4 (DNA ligase 4)	Mus Musculus	up-regulated	Q80SY4
87	Mib1 (E3 ubiquitin-protein ligase Mib1)	Mus Musculus	down-regulatged	Q8BTF7
668	Sdha (Succinate dehydrogenase [ubiquinone] flavoprotein subunit, mitochondrial precursor)	Mus Musculus	up-regulated	Q8K2B3
58	Herc5 hect domain and RLD 5 isoform 2	Mus Musculus	down-regulatged	XP_001478547
234	Tyro3 TYRO3 protein tyrosine kinase 3	Mus Musculus	down-regulatged	ENSMUSP00000106410
729	Gzma Isoform HF1 of Granzyme A precursor (Fragment)	Mus Musculus	up-regulated	P11032-1

### Verification of the Differentially Expressed Hippocampal Proteins by Western Blotting

**Figure 6:**
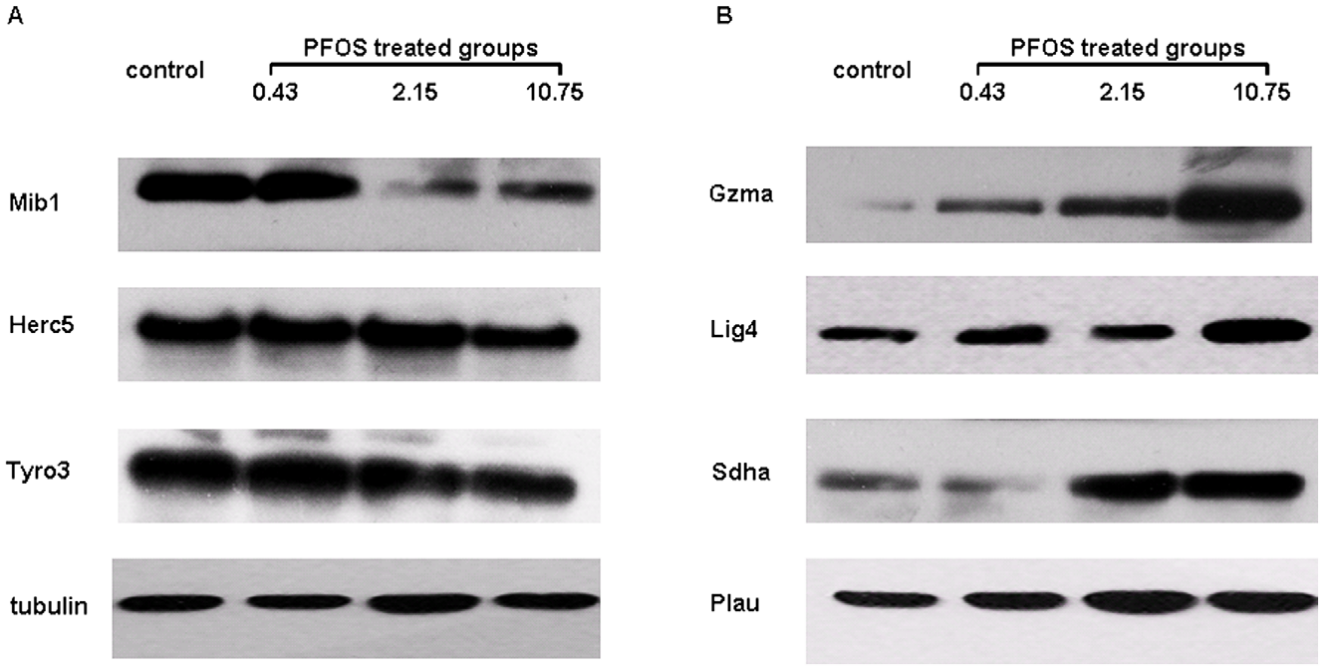
Verification of the differentially expressed hippocampal proteins by western blotting. (A) Compared with the control group, Mib1, Tyro3 and Herc5 were found down-regulated due to PFOS-exposure. (B) Compared with the control group, Lig4, Sdha, Plau and Gzma were found up-regulated due to PFOS exposure.

To further confirm the differentially expressed hippocampal proteins found in 2D-DIGE, we used western blotting analysis which showed the consistent results (Fig. 6), mainly including (i) Mib1, Herc5, and Tyro3protein were found down-regulated in three PFOS-treated groups. (ii) There was significantly increased expression of Gzma, Lig4, Sdha and Plau in 2.15 and 10.75 mg/kg groups. The tubulin protein was used as the internal standard.

## Discussion

In the current study, we have shown that exposure to PFOS leads to the impaired spatial learning and memory, increased glutamate in the hippocampus, slightly decreased DA and DOPAC in the Caudate Putamen of adult mice. Compared with the control group, significant apoptosis of hippocampal cells was found after PFOS exposure, accompanied with the obvious changes of apoptosis related proteins, including the up-regulation of caspase-3 and the down-regulation of Bcl-2, Bcl-XL and suivivin. All these suggested that (i) exposure to PFOS will cause apoptosis in hippocampal cells, and hippocampus might be sensitive to the PFOS neurotoxicity; (ii) the apoptosis occurs hippocampal neural cells will undoubtedly contribute to the impairments of spatial learning and memory after PFOS-exposure; (iii) interestingly, at high dose of PFOS exposure group, in spite of the apoptosis, the endogenous glutamate in hippocampus increased significantly, which in turn, might prompt an excitotoxicity and exacerbate the damage to hippocampus. The slight decrease of endogenous DA in Caudate Putamen indicated the negligibletoxic effects on striatum due to PFOS-exposure. According to the previous study, it is known that DA in the caudate putamen is the neurotransmitter related to the work memory [16], and our results of DA analysis and DA index of turnover in caudate putamen indicate the negligible toxic effects on striatum due to PFOS-exposure.

Furthermore, seven differential expression hippocampal proteins were identified after PFOS-exposure. Among which, Herc5, a proteins with a HECT domain thought to act as E3 protein ligase for ubiquitin [17], was found down-regulated. At the same time, the expression of the E3 ubiquitin ligase Mib1 was down-regulated after PFOS exposure. While, DNA ligase IV (*Lig4*) was found up-regulated. It’s suggested that *Lig4* contributed to repair the particularly deleterious DNA lesion (including DNA double-strand break, DSB) in the developing nervous system [18], therefore, the up-regulation of *Lig4* expression might be a protential protection against PFOS neurotoxicity in the hippocampus. Actually, all these three proteins which response to PFOS exposure have not been reported to specifically related with the dysfunction of hippocampus. Tyro3, another down-regulated protein in our 2D-DIGE result, was believed to play a role in various processes including neuron protection from excitotoxic injury [19].

It’s worth noting that, after PFOS exposure, Plau, an interesting hippocampal protein, is up-regulated. Previous studies have reported, overexpression of Plau during the neuronal development stage is harmful to the hippocampal neurons [20] while it also been found as a potential neuroprotectant for the treatment of acute brain injuries [21]. Herein, we find for the first time the up-regulation of Plau expression caused by high dose PFOS exposure, but further work is needed to discuss whether it is involved in the neuroprotection against neurotoxicity of PFOS. In our study, further work is needed to identify whether the abnormal expression of these identified proteins is related to hippocampus defects due to PFOS exposure. Furthermore, we also found that some apoptosis-related proteins, including caspase-3, Bcl-2, Bcl-XL and survivin significantly changed in hippocampus, which played a role in the apoptosis of hippocampal neural cells due to high dose PFOS exposure.

In conclusion, exposure to PFOS for a long time causes the apoptosis of hippocampal neural cells in adult mice accompanied by the expression alteration of apoptosis-related proteins and the abnormal increase in glutamate in the hippocampus, which leads to the lesions in hippocampus and results in spatial learning and memory defects.

## Materials and Methods

### Animals and Chemical Exposure

Adult C57BL6 mice maintained in the POSTECH animal facility in specific-pathogen-free conditions and under institutional guidelines were used. In our study, a total of 60 mice were divided into 4 groups (n = 15 per group; 8 weeks old). According to the previous studies [11], [12], one group in our study was orally treated with normal saline, and the other three groups were orally exposed to different doses of PFOS: 0.43, 2.15, and 10.75 mg/kg body weight, once/per day for 3 months. All experiments were performed during the light phase of the cycle, with the exception of the water maze studies, which were conducted during the dark cycle.

This study was carried out in strict accordance with the recommendations in the Guide for the Care and Use of Laboratory Animals of the National Institutes of Health. After PFOS exposure, all mice were killed following the National Institutes of Health guidelines for the humane treatment of animals. The protocol was approved by the Committee on the Ethics of Animal Experiments of the University of Nanjing Medical Univeristy and the Safety Assessment and Research Center for Drugs of Jiangsu Province (Permit Number: 2012-0312GU). All surgery was performed under sodium pentobarbital anesthesia, and all efforts were made to minimize suffering.

### Reagents

PFOS (Cat. No.33607, Sigma-Aldrich) was diluted with normal saline. The anti-Mib1 antibody (M5948) was purchased from Sigma-Aldrich. The anti-Herc5 antibody (sc-55837), the anti-tyro3 antibody (sc-1095), the anti-Sdha antibody (sc-27990), anti-Gzma antibody (sc-5510), anti-Lig4 antibody (sc-28232), anti-uPA antibody (sc-6831), the anti-caspase 3 (sc-623), anti-tubulin (sc-55529), anti-Bcl-2 antibody (sc-8274), anti-Bcl-XL antibody (sc-7195) and anti-survivin (sc-8809) antibodies were purchased from Santa Cruz. The propidium iodide (PI) (P4864) and the annexin V-FITC apoptosis detection kit (APOAF-50TST) were purchased from Sigma-Aldrich. DA(Cat. No. H8502), DOPAC (Cat. No. 850217), HVA (Cat. No. H1252), L-glutamate (Cat. No. 49621) and GABA (Cat. No. A2129) were purchase from Sigma-Aldrich. CyDyes for 2D-DIGE were purchased from GE Helthcare. The secondary antibodies were all purchased from Santa Cruz.

### Morris Water Maze Studies

The water maze experiments were carried out as described previously [22], [23]. We tested approximately equal numbers of male and female mice. Our pool is 1.2 m in diameter and a thermoregulated spiral coil keeps the water temperature at 28±1°C. The pool was located in a room uniformly illuminated by a halogen lamp and equipped with various distal cues. Located inside the pool was a removable, circular platform (12 cm diameter), positioned with its top surface 0.5 cm below the water [24].

Each daily trial block consisted of four swimming trials (15 min intertrial interval), starting randomly from each of four starting positions. Mice that failed to find the platform within 2 min were guided to the platform. All mice had to remain on the platform for 15 s before they were returned to their cages [23], [25]. Mean trial escape latency for each mouse was calculated by averaging escape latencies recorded in each set of trials per day. Then in the last day, the platform was removed from the pool, the mice were released in the opposite quadrant, and their search patterns were recorded for 100 s. The movement of the mice is processed by a digital video-tracking device system that calculates, for example, distance from the platform, relative time spent in different areas of the pool and the number of platform crossings.

The behavioural data were analysed with SMART-LD software (Panlab, Barcelona, Spain). The swimming pattern, latency (time to reach the platform), and swimming rate were used to assess the performance during acquisition. The mean trial escape latency for each mouse was calculated by averaging the escape latencies recorded in each set of trials per day. Same method was used in calculating percentage time spent in target quadrant.

### Determination of Endogenous DA and Metabolite by HPLC

The levels of DA, DOPAC and HVA in the caudate putamen were determined by HPLC with electrochemical detection [26]. For monoamine analysis, the brain regions were homogenized in 0.01 M HClO_4_ and centrifuged at 14,000 g for 15 min and supernatants were collected. The data were expressed as micrograms per gram of wet weight.

### Determination of Endogenous Glutamate and GABA by HPLC

Endogenous glutamate and GABA in the hippocampus were measured by HPLC analysis after precolumn derivatization with o-phthalaldehyde and separation on a C_18_ reverse-phase chromatographic column (10×4.6 mm, 3 m; at 30°C; Chrompack, Middelburg, The Netherlands) coupled with fluorometric detection (excitation wavelength, 350 nm; emission wavelength, 450 nm) [27]. Homoserine was used as internal standard.

### Analysis of Hippocampal Protein Level Alterations by 2D-DIGE

The hippocampus protein was extracted as described previously [28]. After quantification, proteins were labeled with CyDyes as suggested by manufactuers. Mixtures of labeled proteins were separated by 2D gel electrophoresis as described [29]. The separated proteins labeled with CyDyes were detected in gels using a 2D-Master Imager (Amersham Biosciences). After detection, the identical labeled proteins migrating to the same 2D spot were quantified based on the corresponding fluorescence intensities, and their molar ratios were calculated using DeCyder Differential In-Gel Analysis software (Amersham Bioscience). Selected 2D gel spots were analyzed for protein identification. The selected spots were excised from 2D gels using Ettan Spot Picker (Amersham Biosciences). Proteins in the excised gel pieces were digested, separated and identified by LC/MS/MS and database search. Each acquired MS/MS spectrum was searched against the NCBI nonredundant protein sequence database (nr.fasta, May 2004), using the SEQUEST software tool. Database search parameters and peptide identification criteria used were as described [30]. The proteins were identified through at least three identified tryptic peptides.

### Analysis of the Apoptosis of Hippocampal Neural Cells by Flow Cytometry

The isolation of hippocampal neural cells was performed as described previously [31]. The cells were washed in 4°C PBS and fixed with 2% paraformaldehyde solution for 20 min at room temperature. After washing, cells were resuspended in 0.5 ml of hypotonic fluorochrome solution containing 50 µg/ml propidium iodide (PI), 0.1% sodium citrate, and 0.1% Triton X-100 to quantitate the cellular DNA content under the permeabilised condition [32]. The cells were washed with PBS and incubated in a solution of 0.5 µg/ml FITC-labelled annexin V. The stained cells were then analysed by flow cytometry (FACScan flow cytometer,CELLQuest Software system,Becton Dickinson, San Jose, CA). Measurement gates were set using the negative controls.

### Detection the Expression of Hippocampal Proteins by Western Blotting Analysis

The hippocampal proteins detected by western blotting were extracted same to 2D-DIGE analysis. In western blotting analysis, the lysates were separated by SDS-PAGE and proteins were transferred to PVDF membranes (Millipore Corporation, MA). After blocking with 5% non-fat milk for 4 h, the transferred proteins were incubated with primary antibodies overnight at 4°C, followed by rigorous washing and then incubated with peroxidase-coupled secondary antibodies. Finally, the protein bands were detected and tubulin was used as internal control. The membrane was then visualised with enhanced chemiluminescence (ECL) kit (Amersham Biosciences).

### Statistics

Data were expressed as means ± SD. Statistical analyses were performed by one-way ANOVA followed by Scheffe test to calculate significance for comparisons between different treatments with the corresponding control. All calculations were performed with Stata Statistical Analysis Software (version 10, StataCorp, USA). Each experiment was repeated at least three times. For all analyses, the criterion for significance was P<0.05.

## Supporting Information

Figure S1 **Profiles of the endogenous glutamate and GABA in the hippocampus by HPLC analysis.** The samples were precolumn derivatizated with o-phthalaldehyde and separation on a C_18_ reverse-phase chromatographic column and coupled with fluorometric detection (excitation wavelength, 350 nm; emission wavelength, 450 nm. Homoserine was used as internal standard.
(TIF)

Figure S2 **Profiles of DA and its metabolites in the hippocampus by HPLC analysis.** The levels of DA, DOPAC and HVA in the caudate putamen were determined by HPLC with electrochemical detection. The data were expressed as micrograms per gram of wet weight.
(TIF)
