# Supplementary figures and images for: Neurotoxicity of Perfluorooctane Sulfonate to Hippocampal Cells in Adult Mice
Source: PLoS One. 2013 Jan 30;8(1):e54176. doi: 10.1371/journal.pone.0054176 (PMC3559704; doi:10.1371/journal.pone.0054176)

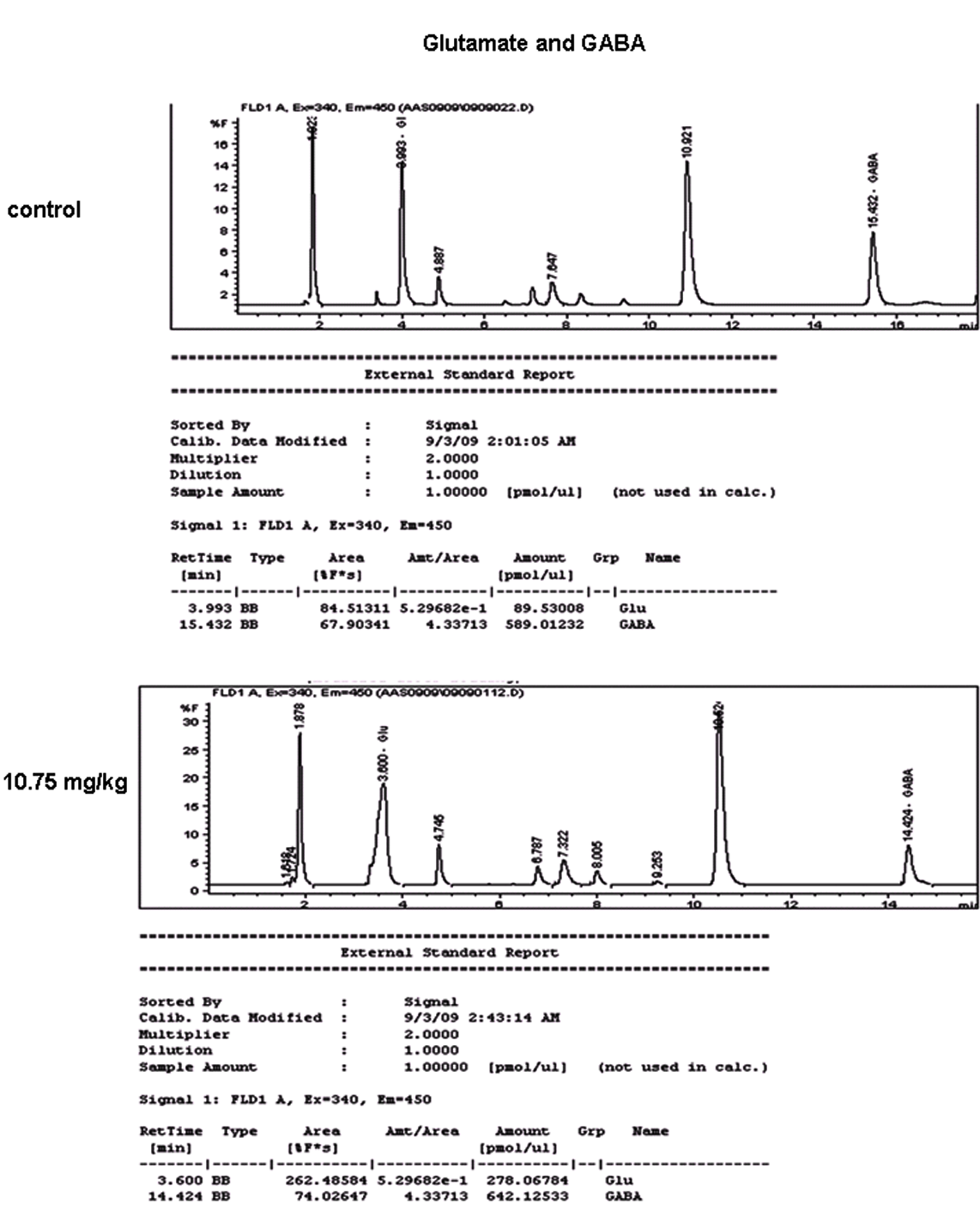

Supplement: Figure S1 — Profiles of the endogenous glutamate and GABA in the hippocampus by HPLC analysis. The samples were precolumn derivatizated with o-phthalaldehyde and separation on a C18 reverse-phase chromatographic column and coupled with fluorometric detection (excitation wavelength, 350 nm; emission wavelength, 450 nm. Homoserine was used as internal standard. (TIF) [file pone.0054176.s001.tif]

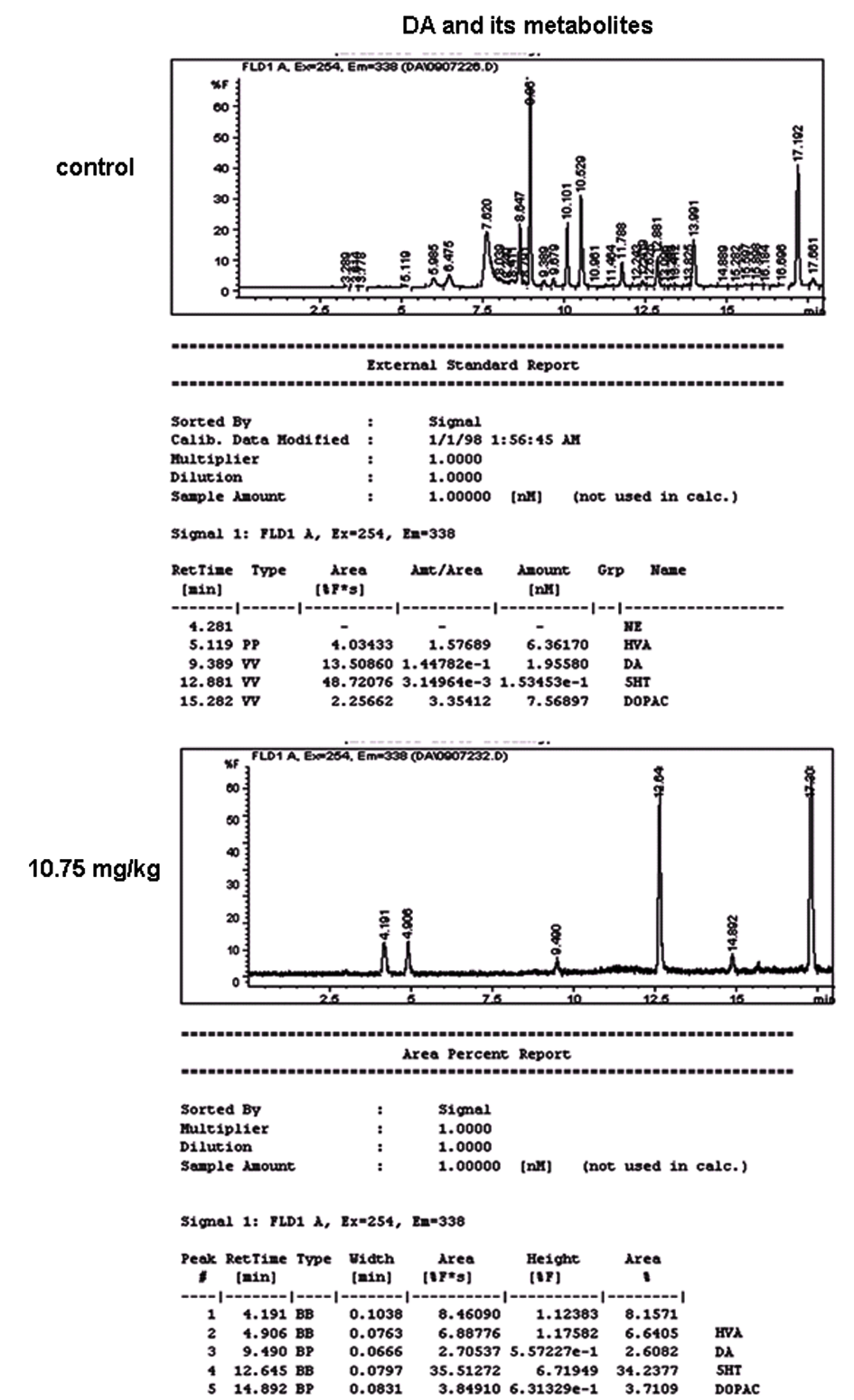

Supplement: Figure S2 — Profiles of DA and its metabolites in the hippocampus by HPLC analysis. The levels of DA, DOPAC and HVA in the caudate putamen were determined by HPLC with electrochemical detection. The data were expressed as micrograms per gram of wet weight. (TIF) [file pone.0054176.s002.tif]
